# Evaluation of microcapillary culture method for the isolation of *Leishmania aethiopica* parasites from patients with cutaneous lesions in Ethiopia

**DOI:** 10.1186/s41512-019-0051-z

**Published:** 2019-03-21

**Authors:** Lensa Aberra, Adugna Abera, Tariku Belay, Amha Kebede, Endalamaw Gadisa, Geremew Tasew

**Affiliations:** 1 Armauer Hansen Research Institute, Malaria and Neglected Tropical Diseases Research Directorate, P. O. Box 1005, Addis Ababa, Ethiopia; 2 Ethiopia Public Health Institute, Malaria and Neglected Tropical Diseases Research Team, P. O. Box 1242, Addis Ababa, Ethiopia; 3 College of Public and Medical Health, Department of Medical Laboratory and Pathology, Jimma University, P. O. Box 378, Jimma, Ethiopia

**Keywords:** Traditional culture method, ITS1-PCR-RFLP, NNN, Diagnosis of CL

## Abstract

**Background:**

In addition to direct slide microscopy, traditional culture method (TCM) has long been considered as a gold standard method for the diagnosis of cutaneous leishmaniasis (CL). However, TCM is relatively expensive and time-consuming compared to the newly introduced microculture method (MCM), which has shown to be sensitive and rapid diagnostic method elsewhere for different *Leishmania* parasite species other than *Leishmania (L.) aethiopica*. The objective of this study was to evaluate the diagnostic performance of MCM for the diagnosis of CL caused by *L. aethiopica.*

**Methods:**

One hundred forty-three lesion aspirates were collected from 124 suspected CL patients prospectively based on their consecutive series. Portion of the aspirates were cultured in duplicate in TCM with modified Novy-MacNeal-Nicolle (NNN) in tissue culture flask and microcapillary tubes containing RPMI 1640 with 10% fetal bovine serum (FBS) for MCM. Smears on glass slides from the remaining portion of the aspirate were used for direct microscopy to detect the parasite after stained with Giemsa staining solution. Up on a consensus, positive result in any two of the three tests was used as a reference standard to analyze sensitivity.

**Results:**

As per consensus standard criteria, 52 of the lesions were qualified to evaluate MCM versus TCM. Forty-eight lesion samples were positive by MCM, 36 by TCM, and 37 by smear microscopy. The representative DNA from parasite culture isolates revealed the causative *Leishmania* parasite was *L. aethiopica* by ITS1 polymerase chain reaction-restriction fragment length polymorphism (PCR-RFLP). Culturing *L. aethiopica* in vitro by MCM is more sensitive (92.3%) than by TCM (69.2%), *P* = 0.003. The median time for *L. aethiopica* promastigotes emergence in the culture was 3 days for MCM and 6 days for TCM, *P* < 0.001.

**Conclusions:**

Our finding indicated that MCM is a sensitive and a rapid culturing method for the isolation of *L. aethiopica* than TCM and smear microscopy.

## Introduction

Leishmaniasis is a vector-borne disease caused by various species of *Leishmania* parasites which are transmitted to mammalian host by the bite of female sand fly. The disease is expressed by various clinical manifestations ranging from self-healing cutaneous lesions to potentially fatal visceral form [1]. Cutaneous leishmaniasis (CL) is a disfiguring skin disease, with the potential of long-term psychological and social consequences, especially in young women [2]. In Ethiopia, the CL form of the disease has got three clinical forms such as localized (LCL), mucocutaneous (MCL), and diffuse CL (DCL) [3].

Cutaneous leishmaniasis is the commonest form of leishmaniasis in Ethiopian highland which is predominantly caused by *Leishmania* (*L*)*. aethiopica*. It is becoming a growing public health concern with increased number of cases and new outbreaks in areas of previously not known to be endemic [4]. It is a zoonotic disease in which the parasites in the ecological system is maintained by rock hyraxes species of *Procavia capensis* and *Heterohyrax brucei*, that have been incriminated as the only known reservoir hosts of *L. aethiopica* [5].

The diagnosis of CL in endemic areas can be made on the basis of clinical and epidemiological data. However, due to potential toxicity associated with standard pentavalent antimonial therapy, identification of the parasites in the clinical specimens is important. Thus, timely and definitive diagnosis is important for the appropriate management of CL. The definitive diagnosis of CL includes visualization of the amastigotes by microscopic examination of Giemsa stained smears or in histological sections and in vitro culture of the parasite [6]. Although microscopic examination is rapid, cheap, and easy to perform, it lacks sensitivity due to the generally low number of parasites found in tissue samples [7]. In vitro cultures obtained from aspirates, biopsies, or from skin scrapings are reported to be more sensitive than microscopy, but the sensitivity is variable and the differences are based on various factors as for example, the viability of collected parasites, the strain and the media used, the presence of super infection, and the expertise of the investigator [8]. Polymerase chain reaction (PCR) is considered as the most sensitive method for the diagnosis of CL [9–13]. However, this method is not yet available outside of the research setting and still remains expensive for field operation. The microculture method (MCM) is reported to be sensitive and has less promastigote emergence time than traditional culture method (TCM) for the diagnosis of CL as described elsewhere [11–16] for CL caused other than *L. aethiopica.*

Cutaneous leishmaniasis induced by *L. aethiopica* is clinically diverse and pleotropic with high genetic diversity as well as resistant to most standard anti-leishmaniasis drugs [17]. Due to such diversity, diagnostic tools working out for other CL may or may not work for the detection of CL due to *L. aethiopica*. Therefore, the objective of this work was to evaluate MCM for isolation of *L. aethiopica* parasite from cutaneous lesions in Ethiopia.

## Materials and methods

### Study area and period

The study was conducted in three health centers (Ankober, Kela, and Kibet) and at All African Leprosy, Tuberculosis and Rehabilitation Training (ALERT) Hospital from April 2012 to February 2013. Ankober health center is located in Amhara Regional State, which serves for 3 towns and 19 administrative kebeles (lowest administrative unit). Kela and Kibet health centers are located in Southern People Nations and Nationalities Regional State. ALERT Hospital is located in Addis Ababa at 7 km southwest. Cutaneous leishmaniasis patients are referred to this hospital from almost all over the country in which they get diagnosis as well as treatment services both at the outpatient and inpatient departments.

### Study population

Patients who were referred to the health center or hospital for suspected CL had a clinical indication for skin scraping or aspirate and were able to provide informed consent before included into the study. Children less than 5 years of age, patients with lesions indicative of inter-current bacterial or fungal super-infection and patients on active treatment for CL were excluded from the study. The operational definition for CL was fulfilled when clinical description that involves appearance of one or more lesions with duration of > 2 weeks, typically on uncovered parts of the body such as the face, neck, arms, and legs were enrolled [5].

Consensus reference standard: defined when a lesion was positive by any two of the three tests (MCM, TCM, and smear microscopy). These tests were served as “reference” standard against which an index test was compared.

### Sample size calculation

Sample size was determined as described previously [11]. The sensitivity and the median time of positivity of TCM and MCM were estimated to be 56% (5.6 + 0.5 days) and 75% (3.5 + 0.5 days) respectively. In order to detect increment in sensitivity of MCM and significant difference in time of positivity, α = 0.05 and power of 80% was assumed to calculate the number of lesions using two population proportion formulas as follows:

z_1 − α_= standard normal *z* values corresponding to the selected alpha

z_1 − β_= standard normal *z* values corresponding to the selected beta

*P*= simple average of the expected proportions

*P*1 and *P*2= expected sensitivity of each method (TCM and MCM)

*n*= number of lesions required in each groups

$$ n=\frac{{\left\{{z}_{1-\upalpha}\sqrt{\left[2P\left(1-P\right)\right]}+{\mathrm{z}}_{1\hbox{-} \upbeta}\sqrt{\left[P1\left(1-P1\right)+P2\left(1-P2\right)\right]}\right\}}^2}{\left(P1-P2\right)2}=\frac{{\left[1.65\sqrt{\left[2\times 0.65(0.35)\right]}+0.84\sqrt{\left[0.56(0.44)+0.75(0.25)\right]}\right]}^2}{\left(0.75-0.56\right)2}+10\%\mathrm{of}\ n=99 $$

Given the above assumptions, the total number of lesions required was calculated to be 99. However, we have collected samples from a total of 143 lesions to compensate for those lesions which were difficult to collect all the three samples specified.

### Clinical sample collection

Each cutaneous lesion of study participants were physically examined by dermatologist for proper identification of the lesion and exclusion of super infection. The lesion aspirates were collected by experienced nurse from individuals who were clinically suspected for CL. Aseptically, lesion aspirates were collected using a 25-gauge needle and disposable syringe containing 0.5 ml of sterile saline (85% NaCl, pH = 7) which was inserted intra-dermally into the outer border of the lesion. The syringe was rotated, and then tissue fluid was gently aspirated into the needle as it is withdrawn. The aspirated fluid was placed in sterile cryotubes which was transported to Armauer Hansen Research Institute (AHRI) Leishmaniasis laboratory from the field sites within cold chain. The aspirated material was divided equally under sterile condition which was then inoculated in TCM or MCM within 5–10 h after collection. Portion of the aspirate at the edge of the needle was used to make a smear on clean glass slide for direct microscopy.

### Direct amastigote detection

Air dried smear on glass slide were fixed by methanol and stained with 10% Giemsa for 25 min. Slides were examined under light microscopy with × 100 objective for detection and quantification of the burden of amastigotes form based on Chulay and Bryceson method [18]. All slides were examined prior to the knowledge of the culture results to avoid subjective interpretation of results and 10% of the slides were confirmed by independent laboratory personnel as part of quality control.

Traditional culture method was performed by using Novy-MacNeal-Nicolle (NNN) medium and Locke’s solution as an overlay media. Whereas for MCM, a commercially available liquid media RPMI 1640 medium (Sigma Chemical Co.) was used with 2 mM L-glutamine and 25 mM Hepes (Sigma Chemical Co.) which was supplemented with 15–20% fetal bovine serum (FBS) (Sigma Chemical Co.) and 100 U/100 μg/ml penicillin-streptomycin (Sigma Chemical Co.). The pH solution was adjusted to 7.2 and filtered through 0.2 μm pore size diameter filter.

### Culturing techniques

Aspirated fluid was inoculated in TCM and the MCM in duplicate and parallel inside bio-safety cabinet under sterile condition. For TCM, 150–200 μL aspirated fluid was inoculated on NNN media immediately after the 3 ml of Locke’s solution was dispensed in the media. The inoculated culture flasks were incubated at 25–26 °C. Whereas MCM was performed by mixing 150–200 μL lesion aspirates with equal volume of complete RPMI 1640 medium in sterile cryo-tube. The mixture was then loaded 2/3 length of 1 × 75 mm non-heparinized microhematocrit capillary tubes (Heinz, Germany) using 1 ml syringe as described previously [19]. The ends of the capillary tubes were sealed and incubated horizontally at 25–26 °C temperature. All inoculated culture flasks and microcapillary tubes were examined every day under an inverted microscope (Leitz-Wetzlar, Germany) with × 20 and × 40 objectives for observation of motile promastigotes in the culture. Cultures were considered positive when a motile promastigote was observed and negative if there was no motile promastigote after being examined for 30 days. The isolates grown to late exponential growth phase were used for DNA extraction and subsequent PCR-RFLP species identification.

### DNA extraction and species typing

The genomic DNA was extracted from culture isolates using commercial kit (QIAamp DNA Mini Kit; Qiagen, Chatsworth, CA, USA) in accordance with the manufacturer’s instructions. Species typing was done using the internal transcriber spacer-1 (ITS1)-RFLP as described by Schonian et al. [20] and Gadisa et al. [21].

### Data processing and statistical analysis

Descriptive statistics was calculated for continuous variables. Categorical variables were quantified by proportions, and statistical analyses were performed using STATA version 11 (StataCorp LP, College Station, TX). The sensitivity, specificity, positive, and negative predictive values of the tests was calculated by using the consensus standard (positive results of any two of three tests) as the “reference standard.” Differences in time to culture positivity were compared between groups by using one-way analysis of variance. Differences in sensitivities were compared using the *z* test. The agreement between two tests was assessed by kappa value, and the level of significance was set as a *P* < 0.05.

### Quality assurance

To check the quality of culture media, positive control cultures was inoculated and treated as the same manner as the clinical isolates. Culture medium with no clinical sample was also used as negative controls. Reference strains (*L. aethiopica* (MHOM/ET/72/L100), *L. donovani* (MHOM/IN/80/DD8), *L. chagasi* (MHOM/BR/00/1669), *L. infantum* (MHOM/FR/LEM-75), *L. tropica* (MHOM/SU/74/K27), and *L. major* (MHOM/ SU/73/5-ASKH) were used as positive controls for species identification and PCR amplification.

## Results

**Fig. 1:**
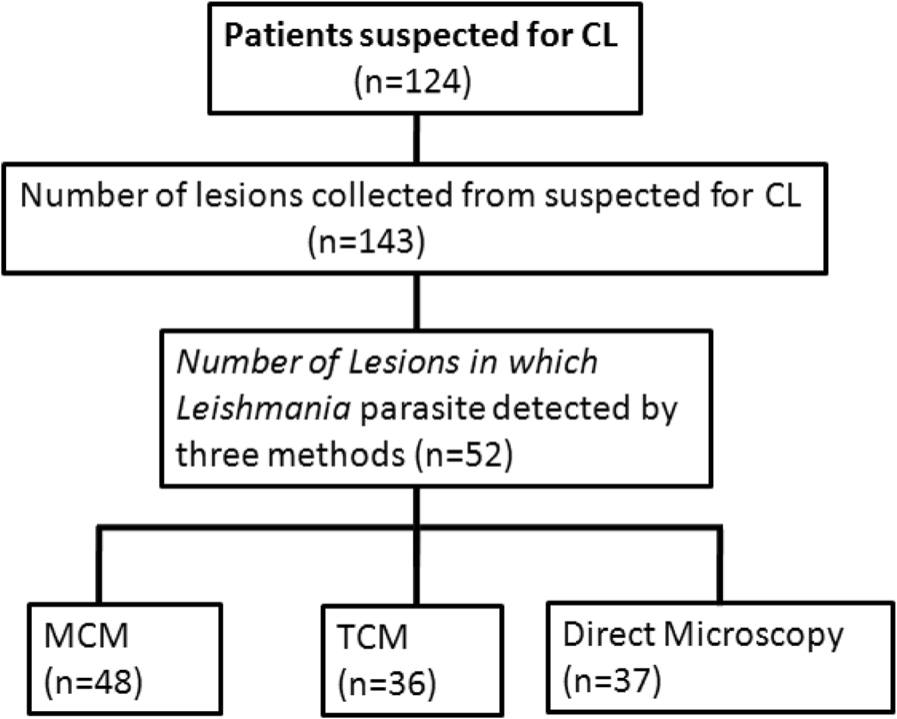
Flow diagram for diagnostic evaluation of MCM for isolation of *L. aethiopica* from CL lesions in Ethiopia

A total of 124 suspected CL cases, of which 38, 33, 38, and 15 of the CL patients were recruited from ALERT Hospital, Ankober, Kela, and Kibet health centers respectively (Fig. 1). Seventy-eight of the study participants were males. From these 124 suspected CL cases, 143 suspected skin lesions were collected and considered as unit of analysis.

The median duration of lesion was 9 months (range 1 month to 20 years). Clinical observation revealed that out of the 143 lesions, 95 (66.4%) were suspected to be LCL, whereas the rest 37 (25.9%) and 11 (7.7%) were assumed to be MCL and DCL respectively. Seventy-six (53.1%) of the 143 lesions were ulcerated while the rest 67 (46.9%) were nodular lesions.

Considering the consensus standard criteria as a reference standard, 52 out of 143 lesions fulfilled the criteria for the diagnosis of CL. Sixty-three lesion samples were positive by at least one of the three tests and 25 were positive by three tests. Of the 52 lesions that were positive by standard criteria, all of them were culture positive by MCM and/or TCM. From these, 48 of the lesions were positive by MCM and 36 of the lesions were positive by TCM, while 37 lesions were positive by direct microscopy (Table 1).

**Table 1:** Clinical characteristics of confirmed CL lesions (*n* = 52) among 143 suspected lesions by CL types, appearance, and lesion duration

Clinical characteristics	Total no. of lesion	Positive parasitological methods			Confirmed CL lesions, n (%)
		MCM, n (%)	TCM, n (%)	Microscopy, n (%)	
CL types					
LCL	95 (66.4)	36 (69.2)	26 (50)	29 (55.7)	40 (76.9)**
MCL	37 (25.9)	3 (5.8)	3 (5.8)	1 (1.9)	3 (5.8)
DCL	11 (7.7)	9 (17.3)	7 (13.5)	7 (13.5)	9 (17.3)
Total	143 (100)	48 (92.3)	36 (69.2)	37 (71.1)	52 (100)
CL appearance					
Ulcerative	76 (53.1)	17 (32.7)	13 (25)	12 (23.1)	20 (38.5)
Non ulcerative	67 (46.9)	31 (59.6)	23 (44.2)	23 (44.2)	32 (61.5)*
Total	143 (100)	48 (92.3)	36 (69.2)	35 (67)	52 (100)
Lesion duration					
< 12 months	108 (75.5)	38 (73.1)	28 (53.8)	25 (48.1)	28 (53.8)
> 12 months	35 (24.5)	12 (23.1)	9 (17.3)	11 (21.1)	24 (46.2)
Total	143 (100)	50 (96.2)	37 (71.1)	36 (69.2)	52 (100)

**P < 0.001 and *P < 0.01. DCL, diffuse cutaneous leishmaniasis; MCL, mucocutaneous leishmaniasis; LCL, localized cutaneous leishmaniasis

From the standard consensus criteria, 76.9% of positive lesions were clinically suspected for LCL (*P* < 0.001), of which 90% were also positive by MCM. When results are compared across lesion appearance, majority of positive lesions were non-ulcerative and the difference was statistically significant (*p* = 0.013).

The overall sensitivity and specificity of MCM was 92.3% (95% [CI] = 84.8–99.8%) and 97.8% respectively. The sensitivity and specificity of TCM was 69.2% (95% [CI] = 54.1–84.3%) and 98.9% respectively (*P =* 0.003) (Table 2). The agreement between MCM and TCM was 83.9% with kappa value of 0.642 (*P* < 0.001). From Table 1 above, it is clear that isolation of *L. aethiopica* parasite from non-ulcerative LCL with lesion duration less than 12 months were best recovered by MCM than TCM and direct microscopy.

**Table 2:** Comparison of performance of three methods for diagnosis of CL from suspected lesions against consensus standard (gold standard, positive = 52, negative = 91)

Diagnostic method	No. of positive	No. of negative	Sensitivity (%)	Specificity (%)	PPV (%)	NPV (%)
Direct microscopy	37	106	71.2	97.8	94.6	84.0
MCM	48	95	92.3^**+†**^	97.8	96.0	95.7
TCM	36	107	69.2	98.9	97.3	84.9

^+^*P* = 0.003 versus TCM by *z* test, ^**†**^*P* = 0.0018 versus direct microscopy by *z* test

When individual patient was used as unit of analysis, the sensitivity of MCM and TCM was 91.1% and 71.1% respectively, while specificity for both MCM and TCM was 98.6%; which did not change substantially from the per-lesion analysis and remained statistically significant (*P =* 0.013).

Thirty-seven lesions were positive by direct microscopy which yielded a sensitivity of 71.2% (95% [CI] = 54.5–80%). There was a moderate agreement (kappa = 0.430) between smear and TCM whereas a substantial agreement (kappa = 0.671) was found between smear and MCM (*P* = 0.0018).

Even though MCM was more sensitive (92.3%) than TCM (69.2%) and direct microscopy (71.2%) in isolation of *L. aethiopica*, all the three tests have similar specificity (Table 2). The three methods have similar in respective of positive predictive value, but MCM (95.7%) has more negative predictive value than the other two methods (Table 2).

### Amastigote to promastigotes transformation is faster in MCM than in TCM

The median time to culture positivity for MCM was 3 days (range 2–11 days) and 6 days for TCM (3–17 days) (*P* *<* *0.001*). When the individual patient was used as unit of analysis, median time to culture positivity did not change substantially from the per-lesion analysis and remained statistically significant (median = 3, range 2–10) while it was 5.5 (range = 3–17) by TCM (Table 3).

**Table 3:** Time to transformation of amastigotes to promastigotes by culture methods

Culturing method	Time to positivity in days (per lesion analysis)			Time to positivity in days (per patient analysis)		
	Mean + SD	Median	Range	Mean + SD	Median	Range
MCM	3.7 + 1.9	3^*^	2–11	3.4 + 1.6	3	2–10
TCM	5.8 + 2.5	6	3–17	5.8 + 2.6	5.5	3–17

^*^P *<* *0.001* by median test

We also compared the costs of MCM and TCM in terms of media consumption per test; sheep blood requirement, autoclaving, and requirement for distilled water. As indicted in Table 4, MCM uses 75 μL of medium per test compared to TCM which uses 4000 μL sheep blood and 2000 μL Locke’s medium per test. The cost for 100 ml of complete RPMI medium is approximately 4.5 USD and the cost of 100 ml Locke’s semi-solid medium is approximately 1 USD. From this comparison, the cost of MCM is at least 5.9 times cheaper than that of TCM.

**Table 4:** Comparison of MCM and TCM media in terms of costs

Comparison	MCM	TCM
Cost of 100 ml sheep blood	Not required	10 USD
Filtration through 0.22 mm	Required	Required for Locke’s solution
Media required/test	75 μL complete media	4 ml sheep blood and 2 ml Locke’s solution
Cost of Locke’s solution/100 ml	Not required	1 USD
Autoclaving	Not required	Required
Distilled water	Not required	Required

The data for comparison was collected from store records, local shops, pharmacy, and laboratories

### Species typing by PCR-RFLP confirms that the infecting parasites were *L. aethiopica*

**Fig. 2:**
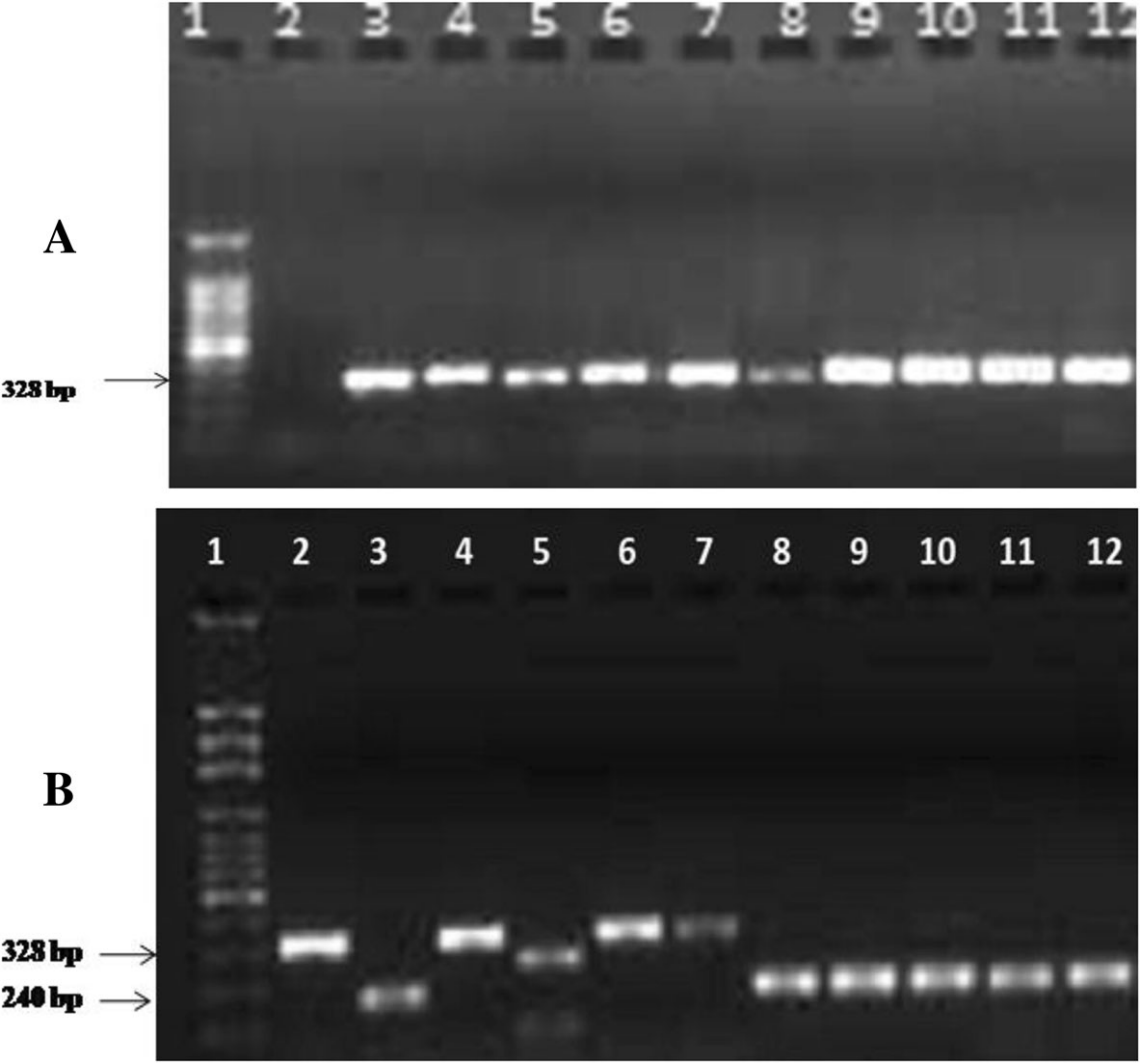
**a** PCR products of ITS-1 from promastigote DNA extract, 1. 100 bp ladder, 2. Negative control, 3. *L. donovani*, 4. *L. aethiopica*, 5. *L. tropica*, 6. *L. major*, 7. *L. infantum*, 8. *L. chagasi*, 9–12 clinical isolates. **b** PCR-ITS1-RFLP of the amplicon with *Hha I*, 1. 100 bp ladder, 2. *L. donovani*, 3. *L. aethiopica*, 4. *L. tropica*, 5. *L. major*, 6. *L. infantum*, 7. *L. chagasi*, 8–12 clinical isolate

Amplification by the ITS-1 primer pairs produced PCR product of about 328 bp (Fig. 2a). When the PCR product was digested by *Hha I* enzyme, *L. aethiopica* reference strain and the DNA isolates from cultured promastigotes formed similar bands approximately 162 bp size (Fig. 2b). This confirms the infecting *Leishmania* parasites were *L. aethiopica*. *L. major* on the other hand yielded two bands of about 88 bp and 240 bp while, *L*. *infantum*, *L. donovani*, *L*. *chagasi*, and *L. tropica* gave single band size of 328 bp.

## Discussion

The conventional CL diagnostic methods currently employed in Ethiopia are slide microscopy and cultivation of *Leishnamia* parasite using TCM. However, utilization of slide microscopy is compromised by lesser sensitivity while TCM is not considered applicable in regional laboratories due to logistic and infrastructure barriers. In present study, we have set a consensus standard criteria in which any two positive tests out of the three tests were considered as a gold standard against which each individual method is compared [22]. In isolation of *Leishmania* promastigotes from CL lesions in the present study, MCM was shown to be more sensitive (92.3%) than the TCM (69.2%) and smear microscopy (71.1%). Microculture method is offering a simpler, cost-effective, and sensitive alternative to TCM and smear microscopy in the diagnosis of CL. In the previous studies, the sensitivity of TCM shown to be varied with parasites load [12], culture media used [16], *Leishmania* species [23] duration, and appearance of lesions [15]. In contrast, the sensitivity of MCM was shown not to be affected by CL clinical categories [15]. The higher sensitivity of MCM in the present report is in agreement with the previous finding (83.3–97%) by Allahverdiyev et al., who also explained the reason to be due to the capillary tube ability to concentrate the sample material and provide microaerophilic conditions favorable for transformation of the amastigotes to promastigotes. In addition, higher partial CO_2_ (pCO_2_) in MCM was reported in the same study which corresponds with the reduction in pO_2_ and pH also favoring the survival of promastigotes [12].

Nevertheless, other studies in Peru reported lesser MCM sensitivity than our finding which is 71.7% [11] and 78.3% [15]. This variation could be attributed to the difference in *Leishmania* species involved in which in Peru the characterized parasite was *L. (V.) braziliensis* complex, different from ours, *L. aethiopica*. However, the lesser median time of MCM positivity reported in our case was in agreement with previous studies, and there is no considerable difference was observed among species [15].

MCM have been shown to be rapid and easy for transportation, and characterization of microcapillary cultivated promastigotes of *L. donovani* [19], *L. tropica*, and *L. infantum* [24]. In the present study, eight MCM (two from each study sites) were mass cultured and characterized by ITS1-RFLP which were identified to be *L. aethiopica*.

Since there is no safe procedure for extraction of cultured promastigotes, we have adapted our own techniques which involve breaking the capillary tubes with care and withdrawing the fluid with fine needle. However, such maneuver has to be evaluated further with care to minimize the risks of laboratory acquired *leishmania* infection.

In terms of medium consumption, MCM uses 75 μL of medium per test compared to TCM which uses 2000 μL medium per test. Similarly, the costs of MCM is at least 5.9 times cheaper than that of TCM, since the cost of 100 ml of complete RPMI medium is approximately 4.5 USD and the cost of 100 ml Locke’s semi-solid medium is approximately 1 USD.

A 100 ml sheep blood and 50 ml of Locke’s overlay solution could be used for 20–25 tests at a total cost of 10.5 USD. Whereas, a 100 ml of complete media could be used for approximately 1333 tests at a total cost of 4.5 USD [25]. Microculture method could not occupy much space in the incubator and easy to transport from place to place compared to TCM. One of the most advantages of MCM is that it can utilize light microscope which could be available at all district levels compared to TCM which utilizes inverted microscope which is only available at higher institutions.

Rapid isolation of CL causative organism in Ethiopia will also facilitate more rapid species identification in institutions where molecular diagnostic capabilities exist. Because the experience from other countries shows that species identification becomes increasingly important due to species variation in response to therapy [26] and in a region where leishmainasis/HIV co-infection is considered high [27]. Inter-specific variability in response to standard anti-leishmaniasis drugs, such as amphotericin B, miltefosine, pentamidine, and paromomycin, has been observed among other species of *Leishmania* parasites somewhere else [26]. In respective of these views, prompt diagnosis and rapid isolation with species identification of CL in Ethiopia are so clinically helpful for dermatologists to treat CL and may contribute in the control and prevention of the disease.

## Conclusion

Our finding indicated that MCM is a sensitive CL diagnostic method than TCM and smear microscopy. As it was consistently shown in other studies, MCM also proved to be economical and rapid culturing technique in our finding as well. Thus, this report depicted the value of MCM for the isolation of *L*. *aethiopica* promastigotes from lesions of CL patients in Ethiopia.

## Abbreviations

AHRI: Armauer Hansen Research Institute
ALERT: All Africa Leprosy, Tuberculosis and Rehabilitation Training
CL: Cutaneous leishmaniasis
DCL: Diffuse cutaneous leishmaniasis
FBS: Fetal bovine serum
ITS-1: Internal transcriber spacer-1
LCL: Localized cutaneous leishmaniasis
MCL: Mucocutaneous leishmaniasis
MCM: Microculture method
NNN: Novy-MacNeal-Nicolle
PCR: Polymerase chain reaction
RFLP: Restriction fragment length polymorphism
RPMI: Roswell Park Memorial Institute
TCM: Traditional culture method
